# A multiplex real-time reverse transcription polymerase chain reaction assay for differentiation of classical and variant II strains of avian infectious bronchitis virus

**DOI:** 10.1007/s00705-022-05603-7

**Published:** 2022-09-29

**Authors:** Sara M. Ameen, Amany Adel, Abdullah Selim, Asmaa Magouz, Mohammed AboElKhair, AbdelHamid H. Bazid

**Affiliations:** 1 Reference Laboratory for Veterinary Quality Control on Poultry Production, Animal Health Research Institute, Agricultural Research Centre, Dokki, PO Box 246, Giza, 12618 Egypt; 2 Department of Virology, Faculty of Veterinary Medicine, Kafrelsheikh University, Kafrelsheikh, 33516 Egypt; 3 Department of Virology, Faculty of Veterinary Medicine, University of Sadat City, Sadat, 32897 Egypt

## Abstract

Identification of avian infectious bronchitis virus (IBV) genotypes is essential for controlling infectious bronchitis (IB) disease, because vaccines that differ from the circulating strains might not provide efficient cross-protection. In Egypt, IBV strain typing is a difficult process, due to the widespread distribution of four genotype lineages (GI-13, GI-23, GI-1, and GI-16), which may contribute to IBV vaccination failure. In this study, we developed a multiplex real-time quantitative reverse transcription polymerase chain reaction (mRT-qPCR) assay that targets highly conserved areas of the S1 gene in order to detect classical (G1) and Egyptian variant II (G23) strains in allantoic fluids and clinical samples. The viral genotyping technique was assessed using commercially available vaccines as well as local strains, and 16 field isolates were tested to investigate its clinical applicability. The assay was found to be specific for the detection of classical and VAR II strains and did not detect the VAR I strain or other avian pathogens such as Newcastle disease virus, avian influenza virus (H9N2 and H5N8), or infectious bursal disease virus. The results also showed that 28 out of 41 samples tested positive for IBV utilizing rt-qRT-PCR targeting the N gene and that 26 out of the 28 positive samples were genotyped by mRT-qPCR targeting the S1 gene, whereas the remaining two samples that were not genotyped were VAR 1 (4/91) and VAR I (793/B). Interestingly, the testing could identify combined infections in one sample, indicating a mixed infection with both genotypes. The real-time RT-PCR assay could detect viral RNA at concentrations as low as 10^2^ EID_50_ /ml for both classical and variant II. This assay is rapid, specific, and sensitive. It appears to be a valuable tool for regular disease monitoring that can be used to differentiate as well as identify viruses.

**Supplementary Information:**

The online version contains supplementary material available at 10.1007/s00705-022-05603-7.

## Introduction

Infectious bronchitis (IB) is a highly infectious viral illness that affects the poultry industry around the world [1]. It causes significant economic damage to the poultry sector, even in areas with no velogenic Newcastle disease viruses or pathogenic avian influenza viruses [2]. The illness manifests itself clinically in a variety of ways, predominantly impacting the respiratory system and causing lesions in the reproductive, digestive, and urinary tracts [3]. Although chickens of all ages can be infected with IBV, younger birds are more susceptible than older ones [4]. The etiological agent of IB is infectious bronchitis virus (IBV), a member of the genus *Gammacoronavirus,* family *Coronaviridae* [5]. IBV has a 27.6-kb positive-sense single-stranded RNA viral genome, which encodes four structural proteins: a spike (S) glycoprotein, an envelope protein, a phosphorylated nucleocapsid protein, and a membrane glycoprotein [6]. The S protein is located on the exterior surface of the envelope of the IBV virion and is cleaved into S1 and S2 subunits by a cellular protease during viral maturation [7]. Determinants of serotype specificity and cell attachment, and epitopes for neutralising antibodies are present in the S1 subunit [8]. The S2 component mediates membrane fusion and connects the S1 protein to the viral membrane.

The S1 subunit exhibits more nucleotide sequence variability than S2, and most of this variability occurs within three distinct hypervariable regions (HVRs) consisting of amino acids 38–67, 91–141, and 274–387 (HVR1, HVR2, and HVR3, respectively) [9]. According to Valastro et al. [10], IBV has been subdivided into six primary genotypes (GI to GVI) in addition to 32 subgenotypic lineages as well as some possible groupings represented as distinct variants according to a classification system based on the S1 sequences.

Four genotype lineages with different genetic and pathogenic features have been reported on chicken farms in Egypt. GI-1 includes the classical wild strains and the vaccine-like strains. GI-23 includes both of the Egyptian variant subgroups (Egy/Var-2 and Egy/Var-1). GI-16, including QX IBV, was first isolated in China and is now found in other Asian countries, Africa, the Middle East, and Europe. GI-13, including the 4/91-like strains, is hypothesized to have been derived from the presently used 4/91 vaccine strain [11].

Genotyping is the most frequently used approach for categorization of IBV strains [12]. Reverse transcription polymerase chain reaction (RT-PCR) is used in molecular analysis to identify viral RNA directly in clinical samples or viruses obtained in a laboratory host system. When using RT-PCR to amplify the IBV S gene, this may be combined with nucleic acid sequencing or restriction fragment length polymorphism (RFLP) analysis in order to determine the virus type [13–17]. Other genotype-specific RT-PCR techniques (real-time or classical) for rapid molecular identification of field variant strains and vaccine strains are also available [18–21].

In Egypt, RT-PCR accompanied by complete or partial S1 gene sequencing has been used to detect and characterise IBV strains [22], prompting us to develop an mRT-qPCR assay that can be used for rapid detection of particular IBV types and can be performed on clinical samples.

## Materials and methods

### Virus strains and clinical samples

Forty-one samples were used to evaluate the assay's applicability for strain typing. In this work, several panels of reference materials as well as clinical samples were employed for analytical specificity testing and validation of the assay. Analytical specificity was assessed using reference strains for four other avian pathogens (influenza A virus H9N2 and H5N8, Newcastle disease virus [NDV], and infectious bursal disease virus [IBDV]), three IB vaccines (MEVAC IB VAR2, AVI IB H-120, and IB 4/91), and nine IBV-negative samples.

Kidneys, lungs, and cloacal and tracheal swabs were among the nine clinical samples taken from broiler, breeder, and layer chicken flocks with clinical signs of IB. In addition, 16 IBV isolates were used to evaluate the performance of the mRT-qPCR assay. The clinical sample collection was obtained in 2021 and stored at -20º C for subsequent processing. The IBV isolates were supplied by the Reference Laboratory for Veterinary Quality Control on Poultry Production (RLQP) and were also isolated in 2021. Details about reference strains of other avian pathogens and vaccines used in this study are shown in Table 1.

**Table 1:** Vaccines and reference strains of other avian pathogens used for testing analytical specificity

Virus	Isolate/vaccine	Origin	Accession no.
IBV	MEVAC VAR II vaccine	MEVAC	
IBV	AVI IB H-120 vaccine	Laprovet-France	
IBV	Var I (4/91) vaccine	Nobilis	
AIV	A/Turkey/Egypt/A2/2021(H5N8)	RLQP-AHRI	OK160062
AIV	A/chicken/Egypt/FAO-S33/2021(H9N2)	RLQP-AHRI	OK148893
NDV	Avian orthoavulavirus 1 fusion protein gene (NDV)	RLQP-AHRI	MZ409479
IBDV	Infectious bursal disease virus segment A VP2 gene (IBDV)	RLQP-AHRI	MZ409478

AIV, avian influenza virus; NDV, Newcastle disease virus; IBDV, infectious bursal disease

### Extraction of viral nucleic acid

Viral RNA was extracted according to the instruction manual of the EasyPure Viral RNA/DNA Extraction Kit (Trans, catalog no. ER201-01). The extracted viral RNA was stored at -20 °C until examination.

### Primer design for multiplex real-time RT-PCR

Probes and primers were designed to distinguish between classical and variant II strains. In addition, customised probes and primers for each genotype were designed based on the most conserved region of the S1 gene of each genotype. Bioedit software (http://www.mbio.ncsu.edu/bioedit) was used to extract and align the S1 gene sequences of IBV classical and variant II strains. The alignment included sequences of five classical genotype field isolates (KC533681, AY135205, KU979009, KJ425497, and DQ487085), 13 variant II genotype field isolates (KU979010, EU780077, JX027070, KU979007, KU979008, KU238171, KU979006, JX173489, KY805846, KC533682, and KC533684, KC533683, and MG233398), and the reference strains Ma5 (KY626045), D274 (MH021175), H-120 (FJ888351), and Mass 41 (GQ504725). The specificity of probes and primers was verified by Basic Local Alignment Search Tool (BLAST) search (www.blast.ncbi.nlm.nih.gov) of the GenBank database. Secondary structures and primer dimers were also predicted using the OligoCalc server (http://biotools.nubic.northwestern.edu/OligoCalc.html.) The sequences of probes and primers used in this study are shown in Table 2. All of the probes and primers were synthesized by Metabion International AG, Germany.

**Table 2:** Sequences of primers and probes used in the multiplex system

Set	Primer ID	Sequence (5´-3')	Position in the spike gene
Variant II	IBV-VAR II-F+	5´-CAA TGG TCC CCG TTT GTG-3´	1128-1145
	IBV-VAR II-R-	5´-GTC TAG GAT GGC TAA ACC AC-3´	1385-1404
	IBV-VAR II-pro+	(HEX) 5´-CCA GGA ATG AAC CAC TTG TGT TAA CTC-3´ (TAMRA)	1235-1261
Classical	IBV-Class-F+	5´-CAT GGT GGT CGT GTT GTT AAT GC-3´	211-232
	IBV-Class-R-	5´- ACA CGT ATA GAA TGC TGT TGA AGC-3´	375-398
	IBV-Class-pro+	(FAM) 5´-CAG GTA TGG CTT GGT CTAGCAGTCAG- 3´ (TAMRA)	263-288

### IBV detection by real-time RT-PCR targeting the N gene

We used real-time RT-PCR targeting the N gene because it has been shown to be a sensitive and accurate method for direct detection of IBV in tracheal or cloacal swabs, as well as in allantoic fluid from infected embryonated eggs [23]. The sequences of the primers and probes targeting the N gene are listed in Table 3.

**Table 3:** Sequences of primers and probes used in real-time RT-PCR targeting the N gene

ID	Sequence	Position in the N gene
AIBV-fr	5´-ATGCTCAACCTTGTCCCTAGCA-3´	811–832
AIBV-as	5´-TCAAACTGCGGATCATCACGT-3´	921–941
TaqMan® probe AIBV-TM	FAM TTGGAAGTAGAGTGACGCCCAAACTTCA- BHQ1	848–875

### Uniplex and multiplex real-time RT-PCR targeting the S1 gene

Real-time RT-PCR was performed using the primers and probes shown in Table 2. Following the instructions of the kit manufacturer, the qRT-PCR mixes were made as follows: 12.5 µl of 2x one-step qPCR mix, 1.25 µl of RT enhancer, 0.25 µl of Verso enzyme mix, 0.5 µl of each of the forward and reverse primers (50 pmol per primer), 0.125 µl of each probe (30 pmol per probe), and 3 µl of RNA, and the total volume of each reaction was adjusted to 25 µl using PCR-grade water. The following thermal profile was used: 15 min at 50°C for cDNA synthesis and 15 min at 95°C for thermo start activation, followed by 40 cycles of denaturation for 15 s at 95°C, extension, and annealing for 1 min at 60°C for standard uniplex IBV detection and 54°C for our multiplex system.

### Analytical specificity of uniplex and multiplex real-time RT-PCR assays

Two sets of primers and probes were designed specifically for distinguishing the strains under investigation and tested and optimized separately on the respective IBV types and then cross-tested for their specificity.

### Analytical sensitivity of real-time RT-PCR

The detection limit of the rt qRT-PCR was determined by testing in duplicate serial tenfold dilutions of the classical IBV vaccine (H120) and variant II IBV strain (F211), which were adjusted to 10^7^ 50% egg infective dose (EID_50_)/ml for both, and standard curves were generated. The detection limit was compared with that of real-time RT-PCR targeting the N gene for both strains. The detection limit was defined as the final dilution at which all tested duplicates could be detected.

### Amplification of the S1 gene of IBV

The genotypes of the viruses in mRT-qPCR-positive samples were determined based on partial sequences of the spike (S1) gene. Briefly, RNA was amplified by conventional RT-PCR, using specific primers for partial identification of the S1 gene [24] (Table 4). RT-PCR was performed using an Easyscript one-step RT-PCR kit (Trans, catalog no. AE411-02) following the kit instructions. Each reaction contained 10 µl of 2x master mix, 1 µl of each primer at a concentration of 20 pmol, 0.4 µl of RT enzyme, 5 µl of each RNA, and PCR-grade water to a final volume of 20 µl. The reaction mixtures were then incubated in a thermocycler with the following thermal profile: 45°C for 30 minutes for the reverse transcription step and 94°C for 15 minutes for RT inactivation, followed by 40 cycles of denaturation at 94°C for 45 seconds, annealing for 45 seconds at 50°C, and amplification for one min at 72°C, and then an extension step for one cycle at 72°C for 10 min.

**Table 4:** Sequences of primers used for conventional RT-PCR for identification of the S1 gene

IBV-F20826	GTTTTATAACTTAACAGTT (RLQP)
IBV-R21298	ATTATAATAACCACTCTGAG (RLQP)

### Partial sequence of spike gene of IBV

A portion of the S1 gene was sequenced by the Sanger method [25]. BioEdit version 7.0 was used for reading the output sequences and for creating a multiple sequence alignment (accessed in October 2021) [26]. The alignment was then used for constructing neighbor-joining phylogenetic trees using the distance-based method in MEGA version 11 [27].

## Results

### A real-time RT-PCR assay targeting the conserved N gene

A real-time RT-PCR that targets the highly conserved N gene was applied to 41 samples to detect IBV, and 28 (nine clinical samples, 16 isolates, and three commercial vaccines) were found to be IBV positive, with C_t_ values between 13 and 31.78.

### Analytical specificity of the uniplex real-time RT-PCR

**Fig. 1:**
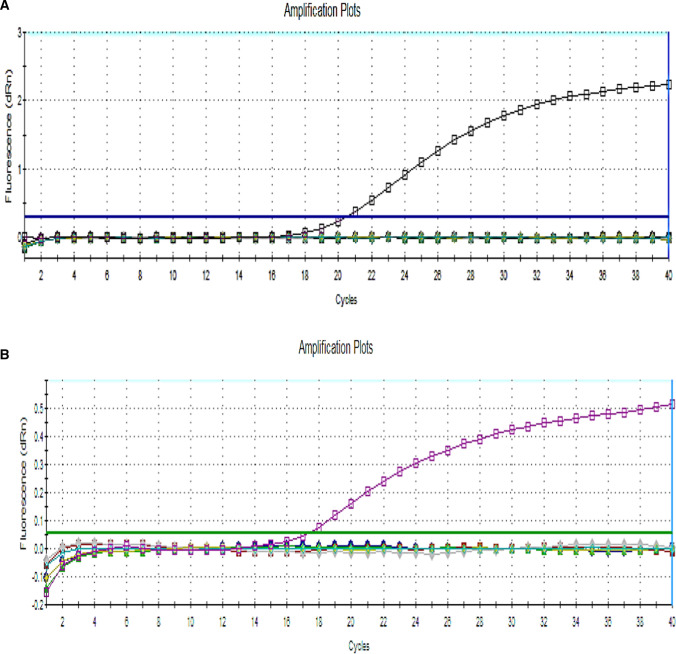
Amplification curves for classical RT-qPCR, showing detection of H120 (C_t_ 20.49) (A), and variant II RT-qPCR, showing amplification of VAR II with sample code F211 (C_t_ 17.29) (B). Neither showed amplification of RNA of H5N8, H9N2, NDV, IBDV, VAR I, or RNA from a negative sample.

The specificity of each probe and primer set was initially examined *in silico* using a BLAST search on the NCBI website. A uniplex RT-qPCR was performed to ensure the workability of each set. Variant II RT-qPCR was able to detect VAR II isolate F211 (GenBank accession no. OK181112), isolate F348, and MEVAC VAR II vaccine, showing no specific amplification of H5N8, H9N2, ND, IBD, VAR I vaccine, H120 vaccine, or the negative control samples, while classical RT-qPCR showed positivity for the H120 vaccine, but it could not detect VAR II isolates (F211 and F348), H5N8, H9N2, ND, IBD, VAR I , MEVAC VAR II vaccine, or the negative control sample. The findings showed that the assay was highly specific with no cross-reactivity (Fig. 1).

### Analytical specificity of the multiplex assay

mRT-qPCR detects several targets in one assay. The assay was able to detect two vaccines (MEVAC VAR II and AVI H120 classical vaccines) but gave negative results for the VAR I (4/91) vaccine and other avian pathogens (H5N8, H9N2, NDV, and IBDV). In addition, nine IBV-negative field samples also showed no specific amplification.

### Analytical sensitivity of the uniplex RT-qPCR

**Fig. 2:**
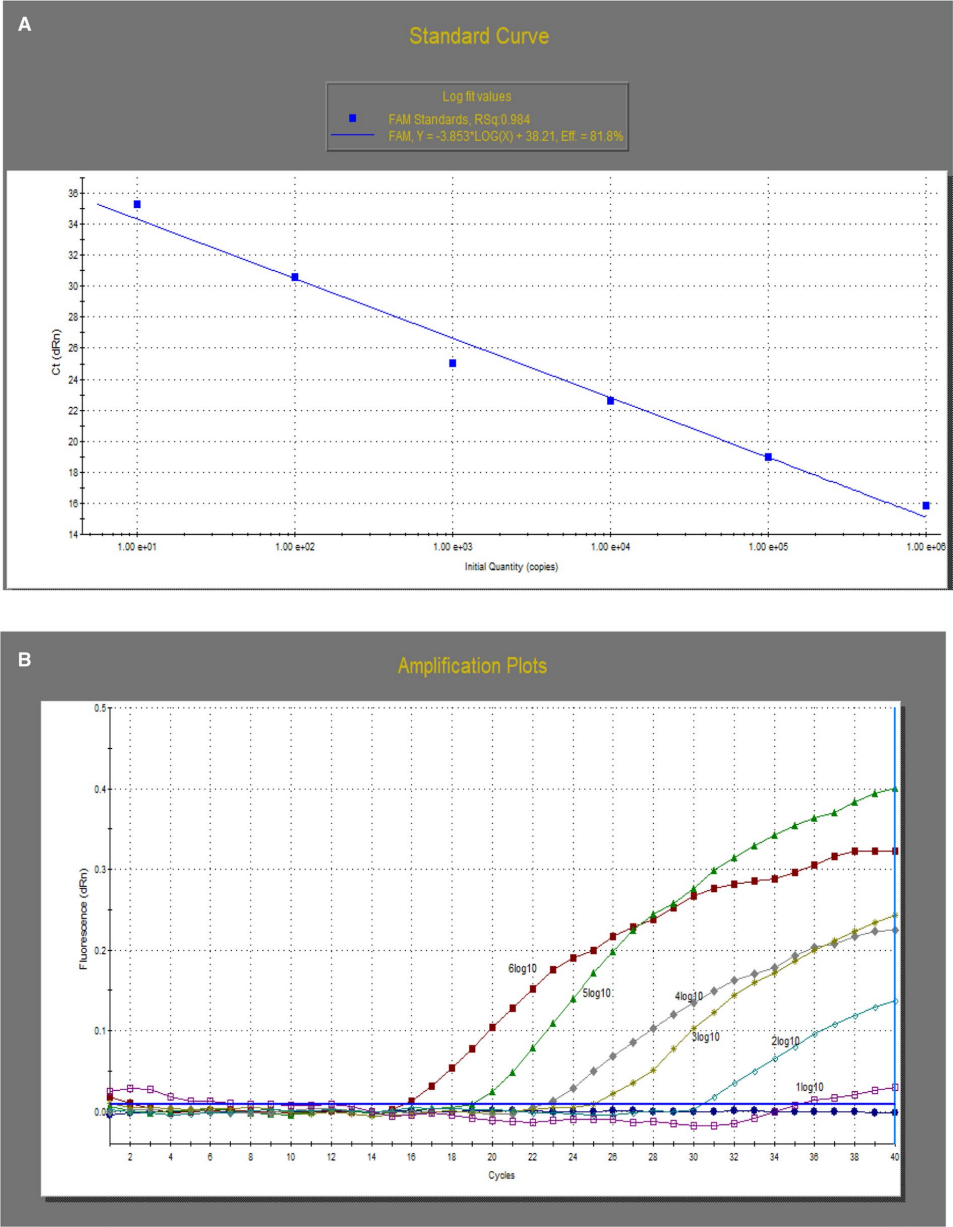

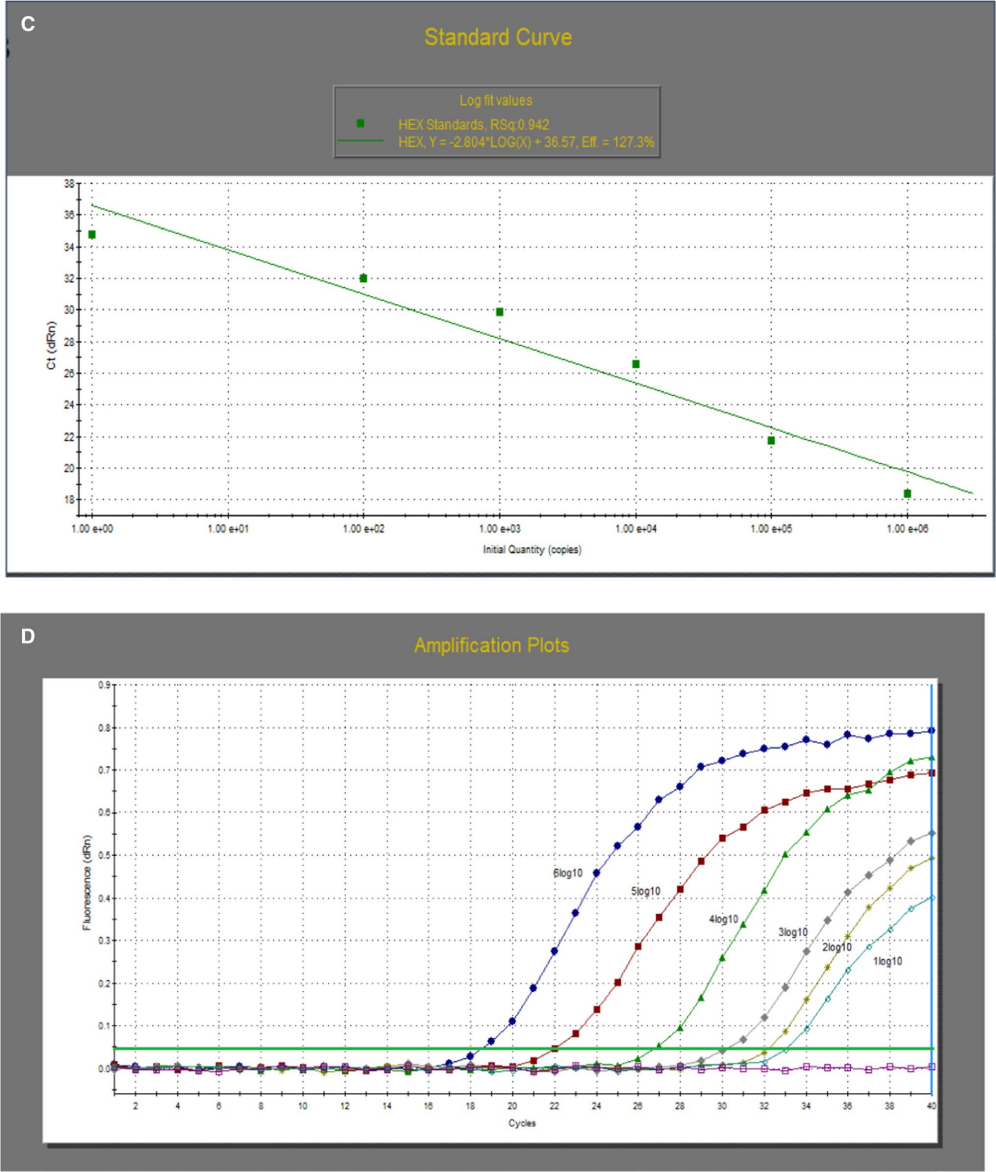
Detection limit and standard curves of the IBV real-time RT-PCR assays for the classical (A, B) and variant II (C, D) genotypes. Each point represents the average C_t_ values for duplicates of each dilution. The coefficient of determination (R^2^) and the amplification efficiency (E) of each assay are indicated.

The average C_t_ values obtained with duplicates of each dilution revealed that the lowest viral load detected by classical RT-qPCR and variant II RT-qPCR for their particular strains was 10^2^ EID_50_ per ml. The two assays showed the same sensitivity as the real-time RT-PCR assay targeting the N gene to detect IBV (Table 5). The standard curves for determining the average copy numbers of the serial dilutions of both classical and variant II reference strains had high R^2^ values (0.98 and 0.94, respectively), as shown in Figure 2, indicating that the use of these primers yielded accurate results.

**Table 5:** Analytical sensitivity of real-time RT-PCR assays targeting the S1 and N genes of classical (H120 vaccine) and variant II (F211) IBV viruses

EID_50_ concentration	F211 (variant II)		H120 (classical)	
	Variant II RT-qPCR (C_t_)^A^	Real-time RT-PCR targeting the N gene (C_t_)^A^	Classical RT-qPCR (C_t_)^A^	Real-time RT-PCR targeting the N gene (C_t_)^A^
10^6^	20.4	19.5	15.7	19.4
10^5^	23.5	23.5	18.7	23
10^4^	26.3	28.2	22.5	27.7
10^3^	29.9	31.5	24.7	29.9
10^2^	32.8	32.4	30.5	33.05
10^1^	33	34.8	35.07	35.7
10^0^	Negative	Negative	Negative	Negative
10^-1^	Negative	Negative	Negative	Negative

^A^C_t_: average cycle threshold

### Applying mRT-qPCR to isolates and clinical samples

**Fig. 3:**
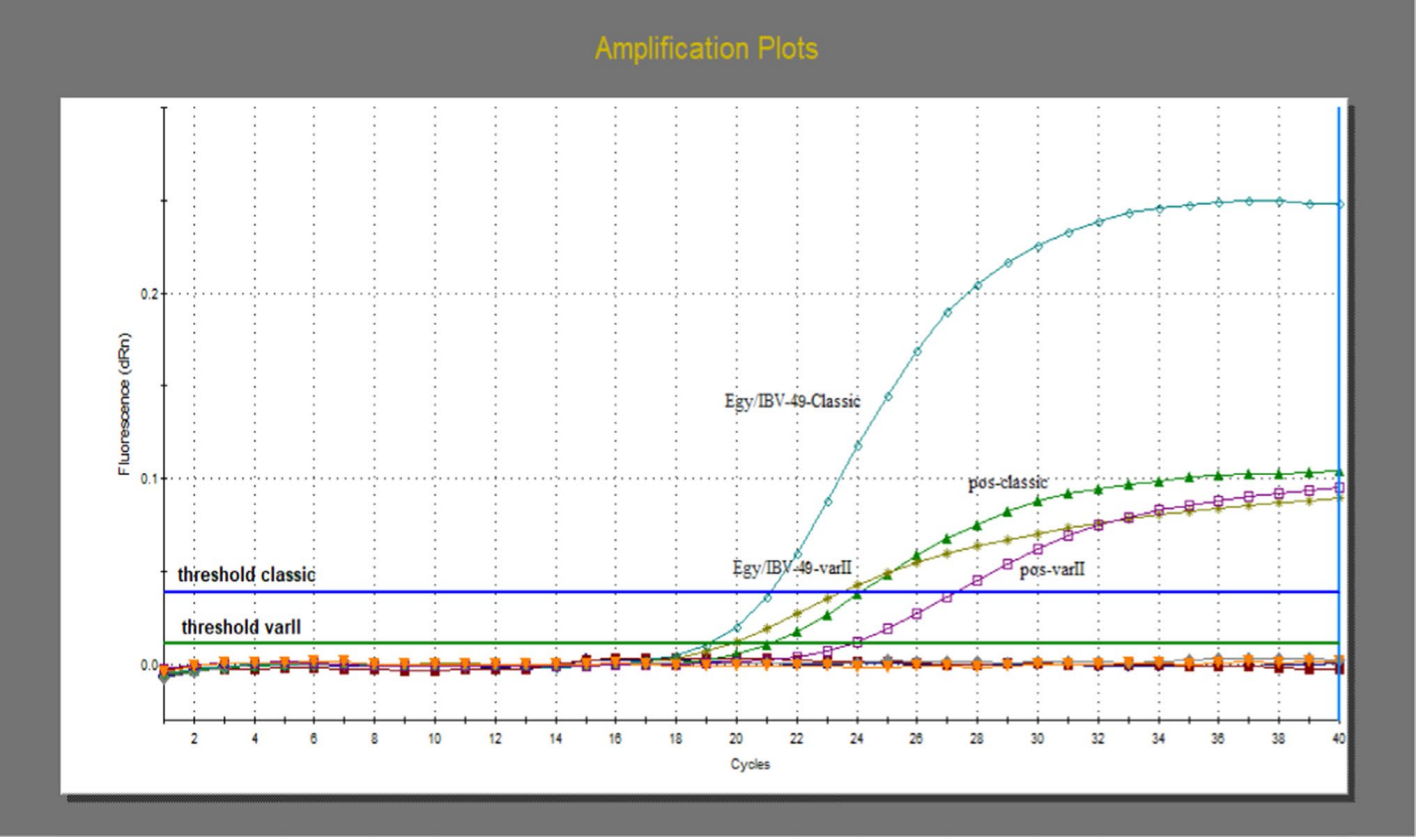
Multiplex RT-qPCR amplification curve for simultaneous detection of classical and Var II in the same sample

The mRT-qPCR assay was carried out on 25 clinical samples and isolates, and the results demonstrated that 22 samples were positive for the variant II genotype (15/16 IBV isolates, 7/9 clinical samples), with C_t_ values ranging from 12.87 to 31. One sample (1/9) was positive for the classical genotype (sample code: 19), with a C_t_ value of 28.24. The assay also detected a mixed infection by both genotypes in one sample (1/16) (sample code: 49), which was positive for classical and variant II genotypes, with C_t_ values of 21.10 and 18.98, respectively (Fig. 3). A field sample previously known as Var I (793/B) (1/9) was tested by mRT-qPCR, and the result was negative (Table 6).

**Table 6:** Comparative C_t_ values of the mRT-qPCR targeting the S1 gene and real-time RT-PCR targeting the conserved N gene in clinical samples and isolates for this study

Sample code		C_t_ (dRn) of the N gene system	C_t_ (dRn) of the VAR II system	C_t_ (dRn) of the classical system
IBV-13	Organs	24	24.12	Negative
IBV-19	Organs	27	Negative	28.24
Egy/IBV-23	Swabs	17.94	20.44	Negative
Egy/IBV-24	Swabs	20.13	22.52	Negative
Egy/IBV-25	Swabs	20.37	23.07	Negative
Egy/IBV-26	Organs	21.68	22.89	Negative
IBV-27	Organs	25.85	24.46	Negative
IBV-28	Organs	22.83	24.15	Negative
IBV-35	Isolate	29.56	28.2	Negative
Egy/IBV-36	Isolate	22.1	21.18	Negative
IBV-37	Isolate	31.14	29.94	Negative
Egy/IBV-38	Isolate	17.48	17.78	Negative
Egy/IBV-39	Isolate	21.47	18.05	Negative
IBV-40	Isolate	31.78	30.58	Negative
IBV-41	Isolate	30.88	31	Negative
Egy/IBV-43	Isolate	23.02	18.68	Negative
IBV-44	Isolate	27.68	26.18	Negative
Egy/IBV-46	Isolate	21.73	18.68	Negative
Egy/IBV-47	Isolate	21.38	20.32	Negative
Egy/IBV-48	Isolate	23.01	21.84	Negative
IBV-49	Isolate	19.92	18.98	21.10
Egy/IBV-50	Isolate	25.72	23.87	Negative
F211	Isolate	13	19.20	Negative
F348	Isolate	14	12.87	Negative
Var I (793/B)	Organs	19	Negative	Negative
MEVAC VAR II	Vaccine	21	21.19	Negative
H120	Vaccine	19	Negative	19.38
Var I (4/91)	Vaccine	19.3	Negative	Negative
H5N8	Reference strain	Negative	Negative	Negative
H9N2	Reference strain	Negative	Negative	Negative
ND	Reference strain	Negative	Negative	Negative
IBD	Reference strain	Negative	Negative	Negative

### Partial sequence of the spike gene of IBV

**Fig. 4:**
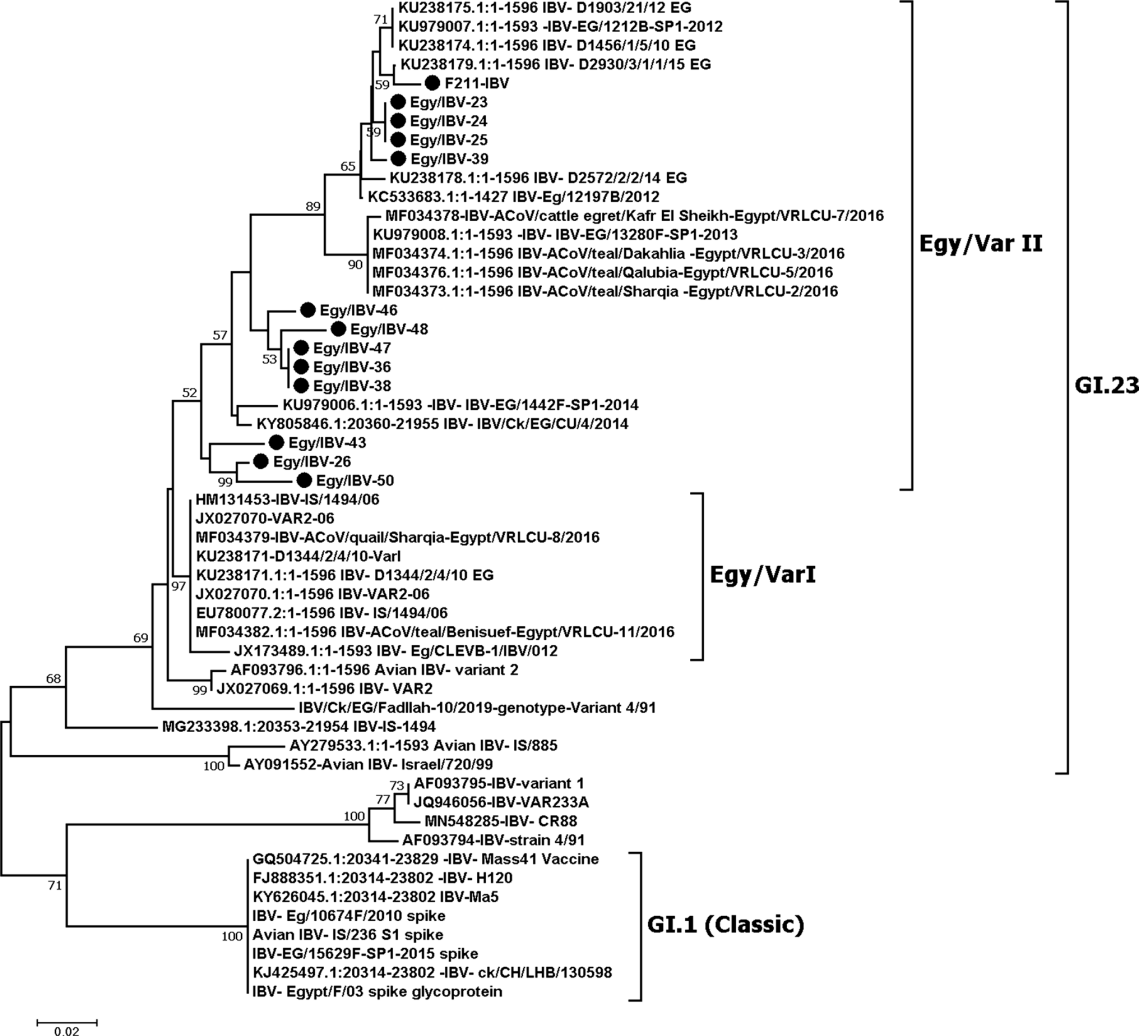
Phylogenetic tree based on partial amino acid sequences of the *S1* genes of 13 IBV clinical samples and isolates (indicated by black dots) and those of other related infectious bronchitis viruses and reference strains.

The amplified RT-PCR products of 13 typed samples (four clinical samples and nine isolates) were purified and sequenced. Sequences produced in this investigation were submitted to the GenBank database, and their accession numbers are listed in Table 7. Phylogenetic analysis demonstrated that the 13 IBV-positive samples clustered with Egyptian variant II sequences (Fig. 4).

**Table 7:** Sequenced IBV isolates and clinical samples and their GenBank accession numbers

Sample code	Sample type	C_t_ (dRn) of the N gene system	C_t_ (dRn) of the VAR II system	C_t_ (dRn) of the classical system	Genotype	GenBank accession no.
Egy/IBV-23	Swabs	17.94	20.44	Negative	VAR II	OL691928
Egy/IBV-24	Swabs	20.13	22.52	Negative	VAR II	OL691929
Egy/IBV-25	Swabs	20.37	23.07	Negative	VAR II	OL691930
Egy/IBV-26	Organ	21.68	22.89	Negative	VAR II	OL691931
Egy/IBV-36	Isolate	22.1	21.18	Negative	VAR II	OL691932
Egy/IBV-38	Isolate	17.48	17.78	Negative	VAR II	OL691933
Egy/IBV-39	Isolate	21.47	18.05	Negative	VAR II	OL691934
Egy/IBV-43	Isolate	23.02	18.68	Negative	VAR II	OL691935
Egy/IBV-46	Isolate	21.73	18.68	Negative	VAR II	OL691936
Egy/IBV-47	Isolate	21.38	20.32	Negative	VAR II	OL691926
Egy/IBV-48	Isolate	23.01	21.84	Negative	VAR II	OL691927
Egy/IBV-50	Isolate	25.72	23.87	Negative	VAR II	OL691937
F211	Isolate	13	19.20	Negative	VAR II	OK181112

## Discussion

IBV causes a severe, highly contagious infectious illness in chickens. IBV is distributed worldwide and causes substantial economic losses in the poultry sector. Genotyping of IBV depends on polymorphisms in the S gene, which codes for the spike protein, particularly in the S1 fragment [3, 10, 28]. S1 gene sequences are highly variable, and mutations in this region may lead to changes in antigenicity [29]. Typing of circulating strains in the field is crucial for selecting appropriate vaccine(s) for controlling the disease [20]. In Egypt, IBV has been spreading among chickens for the past two decades, inflicting massive economic losses to the poultry sector. Numerous IBV genotypes, including GI-23, GI-16, GI-13, and GI-1, have been identified in Egypt, each with unique pathogenic and genetic characteristics [11]. This study presents the development of a multiplex assay for the specific identification of classical (G1) and variant II (G23) IBV genotypes circulating in Egyptian poultry flocks, along with its validation using field samples. The assay was designed based on highly conserved portions of the S1 gene that can be used to distinguish genotypes [20, 21, 30, 31]. This newly designed system was validated using 41 samples (clinical samples, isolates, vaccines, and reference strains of other avian pathogens). A real-time RT-PCR that targets the highly conserved N gene [23] was used as a reference method to evaluate the newly developed real-time RT-PCR assay. All isolates supplied by RLQP, clinical samples collected from birds with clinical signs of IB, and vaccines were positive for IBV, while those collected from birds with no clinical signs of IB and those with other avian viruses were negative.

Two independent real-time RT-PCR assays were conducted to identify classical and variant II genotypes discovered in chickens, and the results showed that the assays were precise with no cross-reactivity. Both assays gave negative results for the VAR I vaccine (4/91) and other avian pathogens, including (H5N8, H9N2, NDV, and IBDV), indicating the specificity of each system. These assays could directly identify and distinguish those genotypes in allantoic fluids and clinical samples. These findings demonstrate the added value of genotype-specific real-time RT-PCR for molecular typing of IBV in Egypt, as was shown in a Brazilian study in which worked Mass RT-qPCR and BR RT-qPCR were developed and validated [31]. Both assays could identify the BR and Mass genotypes in clinical samples and allantoic fluids, allowing easy and rapid molecular typing of IBV. Compared to the uniplex RT-qPCR system, the mRT-qPCR assay reduces both cost and time, since it detects several targets in a single reaction [32, 33]. The analytical specificity of the assay was evaluated by assessing its capacity to identify and distinguish both VAR II and classical genotypes simultaneously. The designed mRT-qPCR was specific for classical and variant II strains, but it could not detect VAR I (4/91) or VAR I (793/B) strains. In addition, there was no cross-reactivity with IBV-negative samples or with samples containing viruses other than IBV. These results indicate that the multiplex assay is specific, as target strains were identified without indication of cross-reactivity between probes and primers, as confirmed by the rRT-PCR assay to distinguish Mass and non-Mass serotypes simultaneously. The assay identified all the strains of IBV among the IBV Mass and non-Mass strains [20].

The detection limit was determined using 10^7^ EID_50_/ml of both classical and VAR II strains as a starting concentration. The assay could identify viral RNA to a dilution of 10^-5^ for both classical and VAR II strains, while the Mass BR RT-qPCR and RT-qPCR, designed to differentiate between BR and Mass genotypes in Brazil, were able to detect viral RNA up to a dilution 2.4 × 10^−6^ [31]. In comparison with the real-time RT-PCR targeting the conserved N gene, classical RT-qPCR and variant II RT-qPCR exhibited almost the same sensitivity. The differences in C_t_ values in the real-time RT-PCR targeting the N gene at the same dilution in two experiments were not significant. The difference did not exceed 0.5, which is an acceptable limit that does not adversely affect the accuracy of the results. This difference might have been due to the handling of the sample during the assay. Meanwhile, the differences in C_t_ values in the Var II vs. classical primer and probes at the same dilution in the sensitivity testing may have been related to the size of the amplicon in each system and its CG content [34]. It has been found that the size of the amplicon in real-time TaqMan probe PCR affects the efficiency of detection. Increasing the amplicon size reduces the sensitivity of detection, thereby increasing the C_t_ value [35].

The newly designed assay could directly identify and distinguish these genotypes in clinical samples. Among the IBV-positive samples, 92% (24/28) were categorized as VAR II strains, demonstrating that the assay is useful for rapid diagnosis of disease. In general, one of the most significant advantages of mRT-qPCR is its potential to identify coinfections. The newly developed assay identified a mixed infection in sample 49 (Fig. 3), which was positive for both genotypes, suggesting a combined infection with the VAR II and classical genotypes. In a previous study [31], a sample with a mixed infection of the Mass and BR genotypes was reported.

Partial S1 gene analysis has been used in previous studies for confirmation of genotype-specific RT-PCR results [19]. In this study, 13 samples were tested by partial S1 gene sequencing, and the results obtained by mRT-qPCR were consistent with those obtained by sequencing and phylogenetic analysis. The isolates from this study are phylogenetically and genetically closely related to Egyptian variant II (13/13, 100%), which indicates the specificity and accuracy of the developed system and agrees with the study of Chen and Wang, who showed that 11 isolates were accurately genotyped by the mRT-PCR assay, in agreement with viral genome sequencing (11/11,100%) [19].

## Conclusion

The mRT-qPCR assay targeting the S1 gene is a promising assay for obtaining rapid, sensitive, and precise results for discriminating between classical and variant II genotypes of IBV.

## Supplementary Information

Below is the link to the electronic supplementary material.

Supplementary file1 (FAS 5 KB)

Supplementary file2 (FAS 3 KB)
